# From cooperation to collapse: the diet-microbiota-host gene triad in disease and aging

**DOI:** 10.3389/frmbi.2026.1872481

**Published:** 2026-07-31

**Authors:** Shreya Bhattacharjee, Arnab Mukhopadhyay

**Affiliations:** Molecular Aging Laboratory, National Institute of Immunology, New Delhi, India

**Keywords:** age-related diseases, aging, diet-gene interactions, diet-microbiota interactions, host-microbe interactions

## Abstract

Symbiotic relationships are the basis of biological complexity. It can be traced back from ancient mitochondrial acquisition to modern host-microbiota interactions. In this review, we explore aging and disease susceptibility through the lens of a diet-microbiota-host gene triad, a dynamic symbiotic network in which dietary inputs, the gut microbiota, and the host genome co-regulate physiological equilibrium. The symbiotic triad evolved as nutrition was outsourced, with dietary and microbial components internalized by the host. Dietary components modulate microbial composition and metabolic activity. In contrast, microbial fermentation of nutrients produces short-chain fatty acids, vitamins, bile acids, and neuroactive compounds, which, in turn, influence host gene expression, immune responses, barrier integrity, nutrient preferences, and health. Host genes have also co-evolved as critical modulators of this triad, encoding nutrient sensors, immune effectors, and proteins that maintain microbial balance and prevent dysbiosis. Polymorphisms in key metabolic and immune genes fine-tune responses to dietary and microbial adaptations, building resilience across different contexts. As organisms age, this triadic equilibrium destabilizes, leading to reduced microbial diversity, compromised barrier integrity and function, and chronic inflammation that accelerates age-related pathologies. Therefore, understanding dietary, microbial, and genetic interdependencies and viewing aging and disease from this perspective offers a blueprint for developing personalized nutrition- and microbiome-targeted therapies to combat age-associated diseases and promote health and longevity.

## Introduction

The evolution of biological complexity is rooted in symbiosis. Life’s first leap toward cooperation occurred when an archaeal host engulfed an alpha proteobacterium, giving rise to mitochondria. These organelles retained their own genome while outsourcing key functions to the host nucleus. These organelles evolved not as subordinates but as metabolic partners, negotiating gene transfer and redox balance to optimize energy efficiency.

This intracellular symbiosis laid the foundation for multicellularity. Over time, interactions expanded beyond the cell, evolving into complex intercellular and organ-level networks. A pivotal shift occurred when organisms began outsourcing nutrient synthesis, relying on external sources, particularly microbes, for essential amino acids, vitamins, cofactors, and neurotransmitters. This metabolic outsourcing enabled reallocation of energy toward advanced traits such as locomotion and neural complexity.

The emergence of the gut- a protected, nutrient-rich internal habitat- transformed host-microbe relationships. Microbes colonizing the gut- bacteria, archaea, fungi, protozoans, and viruses- retained or acquired metabolic functions lost by the host, supplementing the host’s diet and shaping its physiology. Thus, nutrition became a triadic interplay between host, environment, and microbiota. The gut microbiota evolved into a dynamic ecosystem, adapting to dietary pressures and co-evolving with host genetics and physiology.

Gut microbes, by fermenting dietary fiber into short-chain fatty acids and synthesizing vital molecules like vitamins and neurotransmitters, play a crucial role in modulating gene expression, immune responses, and overall health, especially during aging.

This negotiated coexistence formed the basis of “diet-microbiota-host gene interactions”, the molecular dialogues between host genome, microbial metabolism, and environmental inputs. These interactions preserve internal stability amid nutritional variability. In this framework, aging is viewed as a progressive loss of this symbiotic balance. The diet-microbiota-host gene triad destabilizes, leading to dysbiosis and the erosion of metabolic harmony, resulting in inflammation and age-related disease. Understanding diet-microbiota-host gene interactions through the lens of evolutionary symbiosis offers insights into the maintenance of organismal and cellular homeostasis and the mitigation of age-associated decline. While microbiota-host, diet-microbiota, and gene-diet interaction dyads have each been extensively studied and are individually documented throughout this review, the integrative crosstalk among all three of these components has received comparatively less systematic attention. Through this review, we present the concept of the triad and emphasize that it represents an important and necessary component for future research trajectories. The triad represents an important organizing framework that demands future research explicitly designed to examine how interactions between dietary components and host genes shape the microbiota, and vice versa, and how they converge to shape health, disease, and aging.

## Microbe-host interactions: molecular mediators of cooperation and conflict

The body is an ecosystem, inhabited by trillions of microorganisms that colonize diverse anatomical niches, such as the gastrointestinal tract, skin, oral cavity, and reproductive tract. Across the evolutionary timeline, the host and the microbiota have engaged in a genetic arms race that has paradoxically fostered cooperation and a finely tuned, coevolved symbiotic partnership (Sachs et al., 2011; Sharp and Foster, 2022). This interaction operates as a biological dialogue in which host-derived cues, such as gene expression, immune mediators, epithelial secretions, and metabolic signals, shape microbial communities that, in turn, reciprocally influence host genes and physiology, immune homeostasis, lifespan trajectories, and behavior (Sharp and Foster, 2022). Hosts have also evolved regulatory genes that filter microbial metabolites, tolerate symbionts, and suppress opportunists to mount an immune response. The microbiota, in turn, optimize their niche to increase fitness and utilize host resources. This biological entanglement has led to the concept of a hologenome (Rosenberg and Zilber-Rosenberg, 2018), representing the collective genetic repertoire of the host and its microbiota that governs processes such as nutrient metabolism, immune calibration, behavioral regulation, and life history traits (Henry et al., 2021; Ghosh et al., 2022).

Early-life events, such as delivery mode, feeding, antibiotic use, and maternal microbiota, are critical factors that shape initial colonization and long-term microbiome stability. A stable microbiota has immense coding capacity that surpasses even the host, presenting an ever-expanding metabolic potential and acting as a separate “metabolic organ” that derives energy from dietary fibers and carbohydrates, biosynthesizes vitamins, amino acids, and short-chain fatty acids, detoxifies xenobiotic by-products, and even modulates immune responses (Makki et al., 2018; Sonnenburg and Sonnenburg, 2019; Pang et al., 2023).

Various microbial communities and taxa colonize the host throughout its lifetime (**Table 1**). As the host ages, however, this delicate balance of microbiota shifts with multi-level changes, including the expansion of pro-inflammatory taxa, the decline of beneficial strains, weakening of mucosal barriers, and subsequent susceptibility to pathogenic microbes, as well as a chronic low-grade inflammation termed inflammaging (Claesson et al., 2012; Biagi et al., 2016; Kong et al., 2016; Deng et al., 2025). Contemporary research highlights how characteristic alterations in the host’s microbial community constitute a primary hallmark of aging (Wu L. et al., 2022). Centenarian microbiomes exhibit enhanced metabolic capabilities. Functional analyses reveal higher glycolytic potential, short-chain fatty acid (SCFA) metabolism, and alternative amino acid metabolism pathways (Wu et al., 2019). Remarkably, centenarian microbiomes harbor vitamin biosynthesis pathway genes [menaquinone (K2) and riboflavin (B2)], archaeal metabolic genes (coenzyme M and F420), and genes for unique secondary bile acids such as lithocholic acid isomers, which may reduce pathogenic infection risk. Their microbiomes have been found to harbor elevated levels of *Coprococcus* and *Faecalibacterium*, which are taxa associated with anti-inflammatory effects, and also elevated levels of *Akkermansia*, *Lactobacillus*, *Roseburia*, *Bifidobacterium*, *Christensenellaceae*, and *Oscillospira*, which create a metabolic environment enriched in xenobiotic biodegradation pathways, oxidoreductases, and actively combat inflammatory aging processes, while exhibiting decreased abundance of families like *Bacteroidaceae*, *Lachnospiraceae*, and *Ruminococcaceae* (Wu L. et al., 2022; Deng et al., 2025). Centenarians display a unique microbial signature, enriched in potentially beneficial taxa (*Akkermansia*, *Ruminococcaceae*, *Methanobrevibacter*) and metabolites such as indoles and phenylacetylglutamine, which have been linked to extended lifespan in animal models (Sonowal et al., 2017; Wilmanski et al., 2021). These features suggest that microbiome resilience and functional capacity, rather than chronological age alone, may support healthy longevity. Conversely, dysbiosis promotes “unhealthy aging” and is associated with frailty, sarcopenia, osteoporosis, cognitive decline, vascular dysfunction, and immunosenescence. Mechanistically, this may occur through reduced SCFA production, impaired bile acid metabolism, and heightened systemic inflammation (Ticinesi et al., 2020; Brunt et al., 2021).

**Table 1:** Life trajectories of host-microbiota interactions.

Healthy physiology	Microbiota + diet involved	Key metabolites	Mechanisms → host effects	Outcomes (physiology → aging → disease)	References
Core microbiota & colonization	Core taxa (*Actinobacteria*, *Firmicutes*, *Bacteroidetes*, *Proteobacteria*, *Verrucomicrobia*; fungi; archaea); early succession (*Enterobacteriaceae* → *Bifidobacterium* → *Bacteroides*/*Prevotella*/*Ruminococcus*); diet-dependent	SCFAs, vitamins (B, K2), amino acids	Symbiotic metabolic integration; anaerobic succession; immune education	Homeostasis, stable enterotypes → ↓ diversity with age → dysbiosis, developmental imbalance	(Backhed et al., 2015; Costea et al., 2018; Makki et al., 2018; Sonnenburg and Sonnenburg, 2019; Hou et al., 2022)
Development & neuroprotection	Early-life microbiota; SCFA producers; neurotransmitter-producing microbes; fiber diet	SCFAs, neurotransmitters	Morphogenesis; BBB regulation; neuroimmune signaling	Development, cognition → impaired development & neuroinflammation with age → neurodevelopmental disorders	(Reinhardt et al., 2012; Braniste et al., 2014; Kennedy et al., 2018; Silva et al., 2020; Bayer et al., 2021)
Gut-Liver Axis	Dysbiotic microbiota (Western/high-fat diet); SCFA producers (fiber diet)	LPS (lipopolysaccharides), bile acids	TLR4 activation, hepatic inflammation, altered bile acid metabolism	Maintains hepatic metabolism → metabolic dysfunction, increased susceptibility with age → worsened with age and dysbiosis, causing NAFLD, steatosis, cirrhosis, liver disease	(Wang et al., 2016; Kolodziejczyk et al., 2019; Aron-Wisnewsky et al., 2020; Tilg et al., 2021; Pezzino et al., 2022)
Gut-Bone Axis	SCFA producers, *Lactobacillus*, butyrate-producing bacteria, Fiber diet, plant polysaccharides	SCFAs (butyrate), serotonin, osteogenic signals	IGF-1 signaling, osteoblast activation, calcium absorption- Bone formation, mineral absorption	Bone density maintenance, bone strength → bone loss with dysbiosis, increased fracture risk, osteoporosis, bone fractures, reduced bone health	(Zaiss et al., 2019; Chen et al., 2021; Lyu et al., 2023; Hansdah and Lui, 2024; Wei et al., 2025)
Cardiovascular Axis	Dysbiotic microbiota, *Proteobacteria*, Western diet (high-fat, high-sugar, low-fiber)	TMAO (trimethylamine N-oxide), LPS (lipopolysaccharides)	Endotoxemia, TMAO-mediated atherosclerosis, vascular inflammation	Vascular dysfunction, atherosclerosis development, vascular inflammation → Accelerated vascular aging, increased CVD risk, causing CVD, hypertension, atherosclerosis, myocardial infarction	(Koeth et al., 2019; Zhang et al., 2021; Masenga et al., 2022; Jarmukhanov et al., 2024)
Gut-Brain Axis	*Lactobacillus*, *Clostridium*, *Bacteroides*, neurotransmitter-producing bacteria from diet (fiber, polyphenols, probiotics)	Neurotransmitters (GABA, serotonin), SCFA, tryptophan metabolites	Neural signaling, neurotransmitter production, vagal afferent signaling	Cognition, behavior, mood regulation, stress responses → declines in aging: reduced neurotransmitter production, impaired signaling → neuropsychiatric disorders, depression, anxiety, cognitive decline	(Cryan et al., 2019; Chen et al., 2021; Facchin et al., 2024; Loh et al., 2024; Al Noman et al., 2025)
Immune Axis	IgA+ microbiota, T cell-inducing species, innate lymphoid cells	Cytokines, SCFA-derived signals	PRR signaling, TLR pathways, T cell differentiation	Immune homeostasis, tolerance vs. immunity balance, with age: Immune decline, immunosenescence, Autoimmune diseases (dysregulation), infectious disease (reduced immunity)	(Mazmanian et al., 2005; Bouskra et al., 2008; Hapfelmeier et al., 2010; Belkaid and Hand, 2014; Kabat et al., 2014; Zheng et al., 2020)
Immune Tolerance Axis	*Bacteroides fragilis* (PSA+), commensal species	IL-10, polysaccharide A (PSA)	TLR2 signaling, IL-10 production, regulatory T cell induction	Immune tolerance, anti-inflammatory signaling, Th1/Th2 balance, immune tolerance, → dysregulated tolerance, loss of IL-10-producing bacteria, Immune disorders, autoimmune diseases, infection susceptibility	(Rakoff-Nahoum et al., 2004; David, 2010; Erturk-Hasdemir et al., 2019)
Diseased Physiology	Microbiota + Diet Involved	Key Metabolites	Mechanisms → Host Effects	Outcomes (Physiology → Aging → Disease)	References
Dysbiosis & inflammaging	↑ *Bacteroides*/*E. coli*; ↓ SCFA producers; Western/low-fiber diet; *Proteobacteria* expansion	↓ SCFAs; endotoxins; inflammatory metabolites	Barrier breakdown; microbial translocation; LPS signaling; immune dysregulation	Inflammaging, frailty → accelerated aging loop → metabolic and multisystem disease	(Claesson et al., 2011; Claesson et al., 2012; Biagi et al., 2016; Kong et al., 2016; Ticinesi et al., 2020; Brunt et al., 2021; Deng et al., 2025)
Immune & barrier systems	IgA microbiota, T-cell–inducing species; *Lactobacillus*; *Bacteroides fragilis*; IL-22-inducing taxa; fiber/microbial signals	Cytokines, SCFAs, IL-10, antimicrobial peptides	PRR/TLR signaling; T-cell differentiation; IL-23→IL-22 cascade; tight junction regulation	Immune balance, tolerance, barrier integrity → immunosenescence → autoimmunity, infection, IBD	(Rakoff-Nahoum et al., 2004; Mazmanian et al., 2005; Bouskra et al., 2008; Lutgendorff et al., 2008; Anderson et al., 2010; David, 2010; Hapfelmeier et al., 2010; Kinnebrew et al., 2012; Chu and Mazmanian, 2013; Belkaid and Hand, 2014; Kabat et al., 2014; Blackwood et al., 2017; Erturk-Hasdemir et al., 2019; Ren et al., 2020; Zheng et al., 2020)
Neurodegeneration	Dysbiotic microbiota, reduced SCFA producers, Western diet (low-fiber, high-fat), aging	↓ SCFAs, ↑ inflammatory mediators, amyloidogenic compounds	↑ synuclein aggregation, neuroinflammation, oxidative stress	Neural dysfunction, neuroinflammation, protein aggregation → cognitive decline, motor dysfunction, neurodegeneration; Accelerated neurodegeneration, age-dependent onset manifests as Alzheimer's disease, Parkinson's disease etc.	(Yang et al., 2020; Tsamakis et al., 2022; Seo and Holtzman, 2024)
Psychiatric Disorders	Microbial imbalance, dysbiotic taxa, Diet (especially Western diet)	Neuroactive compounds, altered serotonin/GABA pathway	Neuroactive signaling, vagal signaling, immune-mediated neuroinflammation	Behavioral regulation, mood modulation → Worsens with aging, dysbiosis, impaired resilience → Depression, schizophrenia, anxiety, behavioral disorders	(Kochalska et al., 2020; Simpson et al., 2021; Mhanna et al., 2024)
IBD Dysbiosis	↓ *F. prausnitzii, Lactobacillus*; ↑ *E. coli* and pathobionts from Western diet (low-fiber, high-fat)	↓ SCFAs, ↑ inflammatory metabolites (LPS, lipopolysaccharides)	Immune dysregulation, barrier breakdown, TLR activation	Chronic inflammation, barrier dysfunction, immune dysregulation → Worsens with age, difficult to reverse; promotes IBD (Crohn's disease, ulcerative colitis)	(Lloyd-Price et al., 2019; Zheng et al., 2020; Qiu et al., 2022; Talapko et al., 2022; Hanna et al., 2023; Zheng et al., 2024)
Cancer Microbiota	*Fusobacterium nucleatum*, *Helicobacter pylori*, cancer-associated pathobionts, often associated with the Western diet (high-fat, high-sugar, processed)	Polyamines, bile acids, carcinogenic metabolites	Oncogenic signaling, immune evasion, chronic inflammation	Tumor progression, immune evasion→ Increased cancer risk with age, augmented by dysbiosis → Colorectal cancer, gastric cancer, ↑ cancer risk	(Belcheva et al., 2014; Singh et al., 2018; Boehm et al., 2020; Okumura et al., 2021; El Tekle and Garrett, 2023; Chen G. et al., 2024; Fu et al., 2024; Yin et al., 2024; Zeng et al., 2024; Sorino et al., 2025)

### Microbiota’s role in host cellular processes

Microbial metabolism is the primary driver of dietary nutrient metabolism into bioactive molecules, including amino acids, neurotransmitters, peptides, and vitamins, each of which can modulate host gene expression and physiology. SCFAs have emerged as master regulators of host physiology through receptor-mediated and epigenetic mechanisms. By activating GPR41 (FFAR3) and GPR43 (FFAR2), they influence neutrophil chemotaxis, T cell differentiation, and cytokine production (R et al., 2025). SCFAs also cross the blood-brain barrier, modulating neuroinflammation via GPR109A and OR51E2, while regulating intestinal barrier integrity, cytochrome P450 activity, and cardiovascular function (O'Riordan et al., 2022). Butyrate, a master HDAC inhibitor, promotes anti-inflammatory gene expression, Treg expansion, and tolerogenic antigen-presenting cell phenotypes. Early-life SCFA exposure epigenetically programs T-cell development, establishing long-term immune competence. A decline in SCFA-producing bacteria with age is associated with increased inflammation and is linked to autoimmune diseases such as Type 1 Diabetes, rheumatoid arthritis, and multiple sclerosis (Ang and Ding, 2016; Overby and Ferguson, 2021; Kim, 2023; Abdelhalim, 2024; Facchin et al., 2024; Qi et al., 2024; Bui et al., 2025; Liu et al., 2025a). Beyond SCFA, microbiota-specific carbohydrate-active enzymes (CAZymes) help metabolize complex substrates (**Table 2**). Amino acid metabolism, particularly involving tryptophan, bridges microbial biochemistry with host immunity and neurobiology (Wlodarska et al., 2017; Zheng et al., 2023; Bakshani et al., 2025).

**Table 2:** Metabolic potential of host-microbiota interactions.

Metabolic axis	Microbiota + Diet involved	Key metabolites	Mechanisms → Host effects	Outcomes (Physiology → Aging → Disease)	References
CAZyme Metabolism	*Prevotella*, *Bacteroides*, and *Akkermansia muciniphila* obtained from fiber diets, and mucin	Sugars, SCFAs from polysaccharide breakdown	Polysaccharide/mucin degradation, nutrient extraction	Energy homeostasis, nutrient bioavailability → dysregulated in aging, reduced polysaccharide degradation resulting in metabolic disorders (obesity, diabetes)	(Wlodarska et al., 2017; Zheng et al., 2023; Bakshani et al., 2025)
Amino Acid Metabolism	*Lactobacillus*, *Clostridium s*porogenes, and *P*eptostreptococcus from a protein diet	Indoles from tryptophan metabolism, other amino acid-derived metabolites	Tryptophan → AhR signaling, aryl hydrocarbon receptor activation	Immune regulation, mucosal defense, barrier reinforcement → with aging reduced beneficial signaling, barrier compromised, increased inflammation	(Wlodarska et al., 2017; Zheng et al., 2023; Bakshani et al., 2025)
Drug/Xenobiotic Metabolism	Glucuronidases, azoreductases, nitroreductases (enzyme-producing bacteria) from diet and drugs	Modified xenobiotics, drug metabolites	Biotransformation pathways, phase II metabolism	Drug metabolism, detoxification involved in detoxification, drug bioavailability regulation → Age-related variation in enzymatic activity, altered drug metabolism, Drug toxicity variation, altered pharmacokinetics	(Swanson, 2015; Pant et al., 2023; Jyoti and Dey, 2025)
Bile Acid Metabolism	*Bacteroides*, *Clostridium*, *Eubacterium* from a high-fat diet	Secondary bile acids	FXR, VDR, TGR5 signaling, lipid, and glucose metabolism	Metabolism regulation, immune signaling, lipid/glucose regulation assisting in metabolic homeostasis, lipid and glucose control→ Dysregulated with age, altered bile acid signaling, resulting in NAFLD, metabolic syndrome, inflammation	(Peng et al., 2024; Attema and Kuipers, 2025; He et al., 2025; Yang et al., 2025)
Vitamin Synthesis	*Bacteroides*, *E. coli*, *Bifidobacterium*	B vitamins (B2, B12, folate), menaquinone (K2)	Microbial biosynthesis, nutrient bioavailability	Nutritional support, metabolic health → altered with age, increased deficiency risk, Deficiency states, vitamin-responsive disorders	(Kundra et al., 2022; Wan et al., 2022; Tarracchini et al., 2024)

Microbial enzymatic systems also govern the metabolism of xenobiotics and drugs. Over 3,000 microbiome-encoded glucuronidases with distinct substrate specificities, complemented by azoreductases, nitroreductases, and glycyl radical bio-transform pharmaceuticals and environmental toxins, influencing drug efficacy and toxicity (Swanson, 2015; Pant et al., 2023; Jyoti and Dey, 2025) (**Table 2**). Microbial bile salt hydrolases (BSHs) generate secondary bile acids that regulate lipid and glucose metabolism, pathogen resistance, and longevity. At the same time, anaerobic bacteria help expand bile acid chemical diversity by synthesizing microbial conjugated bile acids (MCBAs), which suppress pathogens, promote inflammatory resolution and epithelial regeneration, and support longevity (Swanson, 2015; Peng et al., 2024; Attema and Kuipers, 2025; He et al., 2025; Yang et al., 2025).

Gut microbes share a symbiotic relationship that influences vitamin production, with metagenomic studies revealing age- and geography-specific “vitaminomes”. Microbiome responses to vitamin supplementation vary by donor and diet, influencing metabolite production and metabolic adaptation, and having a profound impact on life-history traits (Kundra et al., 2022; Wan et al., 2022; Tarracchini et al., 2024).

### The epigenome-microbiota crosstalk

The interaction between the gut microbiota and the host epigenome is a fundamentally bidirectional relationship, wherein the microbial metabolites help remodel host chromatin, while epigenetic states in host cells, in turn, influence the composition and functional capacity of the resident microbial community. This reciprocal relationship has now been conceptualized as an ‘epigenome-microbiome axis’ (Pepke et al., 2024).

Short-chain fatty acids (SCFAs), such as butyrate, propionate, and acetate, generated by the anaerobic fermentation of dietary fiber by gut microbes, are among the best-characterized microbial metabolites that act on the host epigenome. Gut commensal microbes produce butyrate at levels sufficient to inhibit histone deacetylases (HDACs) in colonic epithelial cells (Wu et al., 2021). HDAC inhibition leads to increased acetylation of histone H3 and H4 at promoters and enhancers, shifting chromatin towards a more transcriptionally permissive state. In intestinal macrophages, butyrate-mediated HDAC inhibition enhances H3 acetylation at anti-inflammatory loci, represses IL-6 and IL-12 expression, and promotes an M2-like, pro-tolerogenic macrophage phenotype that restrains intestinal inflammation (Woo and Alenghat, 2022).

SCFA-derived acetyl-CoA also serves as a substrate for histone acetyltransferases (HATs), thereby linking microbial metabolism to host-histone modifications (Woo and Alenghat, 2022). Microbiota-derived SCFAs also modulate additional histone modifications, like crotonylation. Antibiotic-mediated depletion of the gut microbiota results in a global reduction of histone H3 lysine 18 crotonylation (H3K18cr) in the colon, implicating microbial products in its maintenance. HDAC1, HGAC2, and HDAC3 have been identified as principal decrotonylases in this context, and their activity has been shown to be inhibited by microbiota-derived butyrate (Fellows et al., 2018).

The gut microbiota also influences DNA methylation through at least two complementary mechanisms. First, commensal taxa such as *Lactobacillus* and *Bifidobacterium* synthesize folate, which fuels one-carbon metabolism and supports the production of S-adenosylmethionine (SAM), the universal methyl donor for DNA and histone methylation. Changes in the microbial community structure can therefore alter SAM availability and reshape the host methylation landscape (D'Aquila et al., 2020). Second, microbial exposure directly modulates the activity of ten-eleven translocation (TET) dioxygenases. Whole-genome bisulfite sequencing in conventionally raised versus germ-free mice has shown that colonization induces localized DNA demethylation at regulatory elements in intestinal epithelial cells in a TET2/3-dependent manner, activating early response genes required for epithelial homeostasis and inflammatory control. In contrast, germ-free mice display widespread DNA hypermethylation in colonic epithelial cells, reduced TET activity, and lowered circulating folate levels, highlighting the dependence of epithelial methylation patterns on microbial signals (Ansari et al., 2020).

Microbial cues also imprint circadian patterns onto the host epigenome. The gut microbiota program diurnal oscillations in histone acetylation marks associated with active promoters, such as H3K27ac and H3K4me, in colonic epithelial cells by driving a rhythmic recruitment of HDAC3 to the chromatin. This rhythmically regulated deacetylation synchronizes intestinal gene expression and metabolic programs with circadian cycles. In germ-free mice, these epigenetic and transcriptional rhythms are markedly dampened or absent, confirming the essential role of the microbiota in the daily epigenetic programming of the intestinal epithelium (Ma et al., 2023).

Conversely, host epigenetic states regulate microbial colonization and spatial organization of the community by controlling the expression of antimicrobial peptides and other barrier components, reinforcing the reciprocal nature of the epigenome-microbiota axis. IEC-specific deletion of HDAC3 results in progressive Paneth cell loss, impaired secretion of antimicrobial peptides, and increased barrier translocation. Notably, these abnormalities manifest only in conventionally raised mice, not in germ-free animals, demonstrating that HDAC3 is required to maintain a stable commensal-host relationship in the presence of microbes (Woo and Alenghat, 2022). In addition, microbiota-induced DNA methylation at the *TLR-4* locus in colonic epithelial cells provides a critical negative feedback mechanism that constrains inflammatory signaling in response to commensal LPS, mediated by the adaptor protein RBM14, which recruits DNMT3 to the TLR4 locus to enforce methylation-dependent repression (Narabayashi et al., 2022; Ferenc et al., 2024).

Perturbations of the microbiota-epigenome axis have been implicated in IBD and colorectal cancer. Dysbiosis-associated loss of butyrate-producing taxa leads to aberrant HDAC activity, altered DNA methylation at inflammatory gene loci, and dysregulation of non-coding RNA networks, including changes in m6A epitranscriptomic marks (Zhang Q. et al., 2025). These insights suggest that interventions targeting this interface, such as probiotic supplementation, prebiotic fiber designed to enhance SCFA production, and HDAC inhibitor-based strategies, may offer mechanistically grounded approaches to restoring epigenetic homeostasis (D'Aquila et al., 2020).

### Microbiota shapes host immune harmony

The commensal microbiota functions as an immunological conductor, orchestrating host defense responses through complex molecular dialogues that go beyond simple pathogen exclusion (Belkaid and Hand, 2014; Zheng et al., 2020). Germ-free animal models highlight their necessity by demonstrating stunted lymphoid architecture, reduced immunoglobulin production, and deficiencies in critical T cells subsets, whereas colonization by commensals restores IgA levels, regulatory and effector T-cell proliferation, and innate lymphoid cell expansion, all in a direct response to microbial cues (Mazmanian et al., 2005; Bouskra et al., 2008; Hapfelmeier et al., 2010; Belkaid and Hand, 2014; Kabat et al., 2014).

Bacterial LPS or flagellin activates the pattern recognition receptor TLR-5 on lamina propria dendritic cells, inducing MyD88-dependent IL-23 production (Chu and Mazmanian, 2013; Zheng et al., 2020). This activates IL-22 in RORγt^+^ ILCs, promoting REGIIIγ secretion, barrier integrity, and pathogen exclusion. Thus, TLR5 deficiency increases susceptibility to colitis (Kinnebrew et al., 2012). Similarly, *Bacteroides fragilis* PSA signals via TLR-2/TLR-1 and Dectin-1 to induce IL-10 and immune tolerance (Rakoff-Nahoum et al., 2004; Erturk-Hasdemir et al., 2019).

### Microbiota as architects of development and tissue integrity

The microbiota is essential for development, morphogenesis, vascularization, epithelial differentiation, and tissue and organ homeostasis, which fundamentally relies on a finely tuned equilibrium between cell death and cellular renewal. Germ-free mouse models exhibit profound morphological aberrations, including diminished intestinal surface area, impaired differentiation of intestinal brush borders, reduced villi thickness, and a characteristic distention of the cecum (Reinhardt et al., 2012; Kennedy et al., 2018; Bayer et al., 2021). Certain *Lactobacilli* species strengthen the epithelial tight junctions, maintaining barrier integrity and rigidity, and promoting proliferation and migration (Lutgendorff et al., 2008; Anderson et al., 2010; Blackwood et al., 2017; Ren et al., 2020) while SCFAs play crucial roles in promoting blood-brain barrier integrity and protecting against neuroinflammation during critical developmental timeframes (Braniste et al., 2014; Silva et al., 2020). Microbe-induced TLR signaling facilitates recovery after intestinal injury, illustrating how immune recognition of commensals correlates with epithelial homeostasis. Dysbiosis disrupts these processes and is closely linked to chronic inflammatory conditions such as inflammatory bowel disease (IBD) and Crohn’s disease, both marked by an exaggerated immune response to commensals and a depletion of beneficial bacteria (**Table 1**) (Lloyd-Price et al., 2019; Zheng et al., 2020; Hanna et al., 2023; Zheng et al., 2024). Reduced SCFAs and accumulation of pro-inflammatory secondary bile acids and polyamines compromise barrier integrity and drive chronic inflammation. Emerging evidence also links oral bacteria to intestinal disease progression, such as the oral *Actinomyces* species that translocate into the intestinal microbiome of IBD patients, highlighting the importance of spatial distribution in microbial architecture (Lloyd-Price et al., 2019; Xia et al., 2025).

### Microbiota in organ homeostasis

Dysbiosis also contributes to metabolic and systemic disorders across organ systems. Dysbiotic microbiomes in the gut-liver axis disrupt hepatic metabolism and inflammation, leading to the development of non-alcoholic fatty liver disease (NAFLD) (Aron-Wisnewsky et al., 2020) through increased *Bacteroidetes* and decreased *Firmicutes*, which enhance lipopolysaccharide translocation (Zhang et al., 2021) and activate hepatic inflammatory cascades via toll-like receptors. Germ-free mice fed high-fat diets exhibit reduced hepatic steatosis compared to conventionally raised mice, whereas SCFA deficiency compromises barrier integrity, facilitating endotoxin translocation and triggering hepatic steatosis; fecal microbiota transplantation studies have shown that these effects can be reversed (Kolodziejczyk et al., 2019; Aron-Wisnewsky et al., 2020).

In skeletal systems, the gut-bone axis integrates gut microbiota with bone metabolism and skeletal health, where SCFAs demonstrate remarkable osteotropic properties, enhancing calcium absorption and osteoblast activity. Recent studies have revealed that diabetic patients exhibit dysbiosis characterized by reduced SCFA-producing bacteria, which contributes to diabetes-associated bone degradation. The microbial modulation of serotonin production, IGF-1 regulation, and signaling has been shown to regulate bone formation and mineral density (Zaiss et al., 2019; Chen et al., 2021; Lyu et al., 2023).

In cardiovascular systems, microbial metabolites, such as trimethylamine N-oxide (TMAO) production, SCFA signaling, and regulation of inflammatory pathways, contribute to hypertension, atherosclerosis, and heart failure by enhancing endotoxin translocation and altering metabolite profiles. Chronic heart failure has been associated with elevated numbers of adherent intestinal bacteria, leading to compromised barrier function and increased cytokine production, ultimately impairing cardiac performance (Zhang et al., 2021; Masenga et al., 2022).

Microbial contributions to cancer are increasingly recognized, with specific microbial taxa directly contributing to oncogenic progression (**Table 2**) (El Tekle and Garrett, 2023). Dysbiotic microbiomes and metabolites, such as polyamines and secondary bile acids, participate in carcinogenic signal transduction. At the same time, altered immune cell recruitment and activation create conditions favorable for tumor initiation and progression (Singh et al., 2018; Boehm et al., 2020; Okumura et al., 2021; El Tekle and Garrett, 2023; Yin et al., 2024).

The microbiota-gut-brain axis is a fundamental regulator of neurological functions, encompassing glial support and function, neurotransmitter production, neurodegeneration, and even neuropsychiatric conditions. Recent studies reveal distinct microbiota diversifications that produce extensive repertoires of neuroactive compounds, including serotonin, dopamine, gamma-aminobutyric acid (GABA), and acetylcholine, which directly influence mood, cognition, and behavior (Cryan et al., 2019; Chen et al., 2021; Loh et al., 2024; Al Noman et al., 2025). Germ-free mice show increased blood-brain barrier permeability, reduced expression of tight junction proteins, deficits in axonal myelination, synaptic maturation, and microglial function, underscoring the necessity of microbes in neurodevelopmental trajectories and enhanced susceptibility to cognitive impairments. Dysbiosis has been implicated in neurodegenerative diseases, such as Parkinson’s, by accelerating α-synuclein aggregation and motor impairment, as well as psychiatric conditions, such as schizophrenia (Tsamakis et al., 2022).

Alzheimer’s disease research has revealed that microbial dysregulation affects neuroinflammation, blood-brain barrier integrity and permeability, and amyloid-β deposition by disrupting communication along the microbiota-gut-brain axis (Seo and Holtzman, 2024). Parkinson’s disease investigations have demonstrated that the gut microbiota influences α-synuclein aggregation in neurons, with specific bacterial populations modulating protein misfolding and subsequent motor function impairment (Yang et al., 2020; Mhanna et al., 2024). Fecal microbiota transplantations have shown significant success in preclinical studies, with clinical trials demonstrating improvements in UPDRS scores, constipation symptoms, and gut microbiota diversity, ultimately leading to better outcomes. These findings also have significant implications for understanding neurodegenerative diseases such as Alzheimer’s and Parkinson’s disease, where gut microbiota dysbiosis contributes to disease progression through enhanced neuroinflammation (Chen et al., 2021; Hashimoto, 2023; Xiong et al., 2023; Facchin et al., 2024; Ma YY. et al., 2024; Ma Z. et al., 2024; Yassin et al., 2025). With aging, microbiota-host interactions shift toward dysbiosis, characterized by a loss in diversity, depletion of beneficial metabolite producers, and expansion of pro-inflammatory taxa in these tissues. This compromises barrier integrity, facilitating microbial translocation into circulation, which sustains low-grade inflammation (inflammaging) and accelerates immunosenescence, thereby creating a feed-forward loop and causing age-related decline (Best et al., 2025).

## Diet-microbiota interactions: nutritional shifts and microbial ecosystem responses

Diet functions as the preeminent modifiable environmental influence shaping the diversity, composition, and metabolic activity of the gut microbiota, a dynamic community whose fluctuations reverberate across both immediate and long-term health consequences (Soldan et al., 2024; Lotankar et al., 2025). Varied dietary regimes orchestrate the gut microbiome’s configuration by modulating bacterial taxa abundance, functional gene expression, and the synthesis of key metabolites, including SCFAs, TMAO, and bile acids (Munteanu and Schwartz, 2024; Lotankar et al., 2025). Microbial biochemistry influences energy balance, immune homeostasis, intestinal barrier integrity, and the inflammatory milieu, establishing diet-microbe crosstalk as a focal point for chronic diseases such as obesity, diabetes, cardiovascular pathologies, inflammatory bowel disease (IBD), neurodegeneration, and even cancer (Croci et al., 2021).

### The effects of high-fiber diets on the gut microbiota

Dietary fibers are an intricate matrix of nondigestible carbohydrates, including cellulose, hemicellulose, oligosaccharides, and resistant starch, which resist enzymatic digestion in the upper digestive tract and arrive intact in the colon, becoming the principal fermentative substrate for the resident microbiota (Senés-Guerrero et al., 2020; Oliver et al., 2021; Thomson et al., 2021). Upon microbial fermentation, the major end products are SCFAs, which fulfill about 2-10% of daily human energy requirements. Their functions are multi-layered: butyrate nourishes colonocytes and confers anti-inflammatory, anticarcinogenic, and immunomodulatory benefits; acetate, the most abundant SCFA, supports lipid biosynthesis and serves as a metabolic substrate for peripheral tissues; propionate participates in hepatic gluconeogenesis and orchestrates appetite and lipid metabolism (Oliver et al., 2021; Facchin et al., 2024).

Fermentable fibers, such as inulin, fructooligosaccharides (FOS), galactooligosaccharides (GOS), resistant starch, and β-glucans, exert pronounced prebiotic effects, promoting the growth of butyrate-producing bacteria (Jangid et al., 2022; Munteanu and Schwartz, 2024; Wu et al., 2025). The consequences of increased fiber intake include enhanced microbial diversity, a vital marker of ecosystem resilience, especially evident in rural or non-Western agrarian communities reliant on unprocessed, plant-based foods. Studies comparing African children on high-fiber diets with European children on fiber-poor diets documented elevated *Prevotella* levels. They enhanced SCFA synthesis in the former, indicating a healthier microbial architecture (De Filippo et al., 2010). Conversely, the Western dietary pattern, marked by fiber scarcity, depletes those beneficial fermenters and suppresses SCFA synthesis, thinning the mucus layer, elevating intestinal permeability, and increasingly exposing the host to infection and inflammatory assault as barrier and immune functions decline (Senés-Guerrero et al., 2020; Thomson et al., 2021; Jangid et al., 2022; Dmytriv et al., 2024; Munteanu and Schwartz, 2024). Interestingly, striking inter-individual differences exist in the magnitude and nature of microbiota and SCFA responses to fiber interventions, which are attributed to baseline microbiota composition, habitual fiber intake, and genetic and environmental factors, discussed later (Lampe et al., 2013; Leshem et al., 2020).

### Metabolic recalibration with ketogenic diets

The ketogenic diet (KD), a high-fat, very low-carbohydrate, and moderate-protein regimen, shifts substrate metabolism towards fatty acid β-oxidation and hepatic ketogenesis, increasing circulating ketone bodies (acetoacetate, β-hydroxybutyrate, acetone) while altering peripheral and intestinal metabolic environments (Santangelo et al., 2023; Tang et al., 2025). In animal models and clinical contexts alike, KD consistently narrows microbiome diversity, notably eroding alpha diversity metrics (Chao1, Shannon index), and decreases Bifidobacterium abundance, a direct consequence of low fermentable carbohydrate availability and the resultant impaired SCFA biosynthesis (Santangelo et al., 2023; Ross et al., 2024; Lotankar et al., 2025; Tang et al., 2025). The KD-induced loss of butyrate producers (e.g., *Roseburia*, *Eubacterium rectale*) and the depletion of SCFAs, especially butyrate and propionate, raise concerns about long-term adverse shifts and the risk of dysbiosis. While short-term KD improves weight management and metabolism among overweight or epileptic patients, it is shadowed by increased gut permeability, reduced fecal SCFA concentrations, and a less robust, more homogeneous microbiota that predisposes to colitis, inflammatory, and metabolic disease in susceptible populations (Zhu et al., 2022; Patikorn et al., 2023).

### Mediterranean diets and microbial enrichment

The Mediterranean Diet (MD) represents a nutritional paradigm, distinguished by abundant consumption of vegetables, fruits, legumes, whole grains, nuts, seeds, and olive oil (particularly, extra virgin olive oil-EVOO), moderate amounts of fish, poultry, dairy, and wine, while minimizing red and processed meats, saturated fats, and sugar. Longitudinal and interventional research consistently demonstrates that MD correlates with diminished incidence of metabolic syndrome, type 2 diabetes, hypertension, and obesity, alongside reduced prevalence and improved progression of IBD, NAFLD, and neurodegenerative conditions, while enhancing cognitive function and mitigating frailty in elderly populations. However, sustained commitment to MD principles appears necessary to ensure durable microbiota restructuring and clinically significant health benefits (Kimble et al., 2023; Khavandegar et al., 2024; Garrido-Romero et al., 2025). MD adherence promotes greater microbial richness and evenness, thereby expanding populations of SCFA-producing bacteria (**Table 3**). The landmark PREDIMED study (randomized controlled trial) reaffirms these effects, demonstrating that MD adherence improves cardiovascular risk profiles, increases gut microbial diversity, and elevates fecal SCFA concentrations (Guasch-Ferre et al., 2017). A lower intake of red meat, choline, and carnitine-rich foods, and interspecies competition by fiber-degrading microbes lead to lower TMAO production, thereby lowering cardiovascular disease risk (Simo and Garcia-Canas, 2020; Li J. et al., 2022). MD also enhances polyphenol metabolism, producing bioactive compounds with antioxidant, neuroprotective, and anti-inflammatory effects that benefit cognitive and vascular health (Khavandegar et al., 2024; Perrone and D'Angelo, 2025). MD improves bile acid signaling, with downstream effects on energy balance and metabolism (Olalekan et al., 2024; Perrone and D'Angelo, 2025).

**Table 3:** Diet–microbiota interactions: composition, metabolism, and health outcomes.

Diet type	Key dietary features	Microbiota changes	Key metabolites produced	Mechanisms / metabolic effects	Health outcomes	Disease associations	References
High-fiber diet	Non-digestible carbohydrates (cellulose, hemicellulose, resistant starch, inulin, fructooligosaccharides (FOS), galactooligosaccharides (GOS), β-glucans)	↑ *Faecalibacterium* *prausnitzii*, *Roseburia*, *Eubacterium rectale*, *Bifidobacterium*, *Lactobacillus*, *Prevotella*	SCFAs (butyrate, acetate, propionate), lactate	Fermentation → SCFAs; butyrate fuels colonocytes; acetate supports lipid metabolism; propionate regulates gluconeogenesis and appetite	↑ Microbial diversity, improved barrier integrity, anti-inflammatory effects	Reduced risk of metabolic disease, inflammation	(De Filippo et al., 2010; Senés-Guerrero et al., 2020; Losno et al., 2021; Oliver et al., 2021; Thomson et al., 2021; Jangid et al., 2022; Facchin et al., 2024; Munteanu and Schwartz, 2024; Lotankar et al., 2025; Wu et al., 2025)
Low-fiber / Western diet (fiber-deficient aspect)	Low fiber intake	↓ SCFA producers; ↑ *Akkermansia* *muciniphila* (mucin degraders)	↓ SCFAs	Reduced fermentation → mucus degradation → increased gut permeability	Impaired barrier, reduced immune function	Infection susceptibility, inflammation	(Senés-Guerrero et al., 2020; Thomson et al., 2021; Jangid et al., 2022; Dmytriv et al., 2024; Munteanu and Schwartz, 2024)
Ketogenic diet (KD)	High-fat, very low-carb, moderate protein	↓ *Bifidobacterium*; ↓ *Roseburia*, *Eubacterium rectale*; ↑ *Firmicutes*; ↓ *Bacteroidetes*, *Actinobacteria*; ↑ *Proteobacteria* (*Escherichia*, *Klebsiella*, *Listeria*); ↑ *Akkermansia*	Ketone bodies (acetoacetate, β- hydroxybutyrate, acetone); ↓ SCFAs	Ketogenesis; reduced fermentable substrate → ↓ SCFA production; altered microbial composition	Short-term metabolic benefits, weight loss in overweight patients	Dysbiosis, colitis risk, inflammation, metabolic disorders	(Santangelo et al., 2023; Guzey Akansel et al., 2024; Li et al., 2024; Ross et al., 2024; Lotankar et al., 2025; Tang et al., 2025)
Mediterranean diet (MD)	High fiber, polyphenols, olive oil, plant-based foods; low red meat	↑ *Bifidobacterium*, *Roseburia*, *Faecalibacterium* *prausnitzii*, *Lactobacillus*, *Prevotella*, *Bacteroides*, *Parabacteroides*; ↓ pathogenic taxa	↑ SCFAs; ↓ TMAO; polyphenol-derived metabolites	Enhanced fermentation; improved bile acid metabolism; reduced TMAO; polyphenol metabolism; immune modulation (↑ Treg); satiety peptide release, increased SCFAs improve mucosal barrier integrity, regulate lipid and glucose metabolism	Improved metabolic health, cognition, lowered systemic inflammation	Reduced CVD, obesity, diabetes, IBD, NAFLD, neurodegeneration	(Guasch-Ferre et al., 2017; Ghosh et al., 2020; Simo and Garcia-Canas, 2020; Li J. et al., 2022; Kimble et al., 2023; Facchin et al., 2024; Khavandegar et al., 2024; Olalekan et al., 2024; Garrido-Romero et al., 2025; Perrone and D'Angelo, 2025; Theis et al., 2025)
EVOO (Mediterranean component)	Olive oil, phenolics (hydroxytyrosol, tyrosol), MUFAs	↑ *Akkermansia muciniphila*, *Lactobacillaceae*, *Bifidobacteriaceae*; ↓ *Firmicutes*/ *Bacteroidetes* ratio	Polyphenol-derived metabolites	Modulates microbial composition; enhances barrier and immune function	Cardioprotective, anti-inflammatory	Reduced metabolic disease risk	(Garrido-Romero et al., 2025)
Vegetarian / Vegan diet (VD)	High fiber, polyphenols; low fat and protein	↑ *Bacteroidetes*, *Prevotella*; ↑ *Faecalibacterium*, *Roseburia*, *Lachnospira*, *Coprococcus*; ↑ *Akkermansia*; ↓ *Enterobacteriaceae*, *Proteobacteria*, *Clostridiales*	SCFAs (butyrate, acetate); ↓ BCFAs, ammonia; ↓ toxic metabolites (p-cresol, indoxyl sulfate); polyphenol metabolites	Enhanced carbohydrate fermentation; reduced protein fermentation; polyphenol biotransformation	Reduced inflammation, improved metabolic profile, lower BMI	Reduced cardiometabolic disease, colon cancer risk (with nutrient caveats)	(David et al., 2014; Tomova et al., 2019; Verhoog et al., 2019; Croci et al., 2021; Losno et al., 2021; Rodriguez-Daza et al., 2021; Trefflich et al., 2021; Cano et al., 2024; Soldan et al., 2024; Lotankar et al., 2025; Wu et al., 2025)
Western diet (overall)	High fat, sugar, processed foods, low fiber, UPFs	↓ *Faecalibacterium*, *Roseburia*, *Eubacterium rectale*, *Bifidobacteria*; ↑ *Proteobacteria*, *Bacillota*, *Pseudomonadota*	↓ SCFAs; ↑ TMAO; ↑ secondary bile acids; ↑ endotoxins	Reduced fermentation; increased permeability; endotoxemia; pro-inflammatory signaling	Reduced microbial diversity, barrier dysfunction	Obesity, CVD, IBD, cancer, metabolic disease	(Simo and Garcia-Canas, 2020; Croci et al., 2021; Gill et al., 2022; Jangid et al., 2022; Li J. et al., 2022; Dmytriv et al., 2024; Soldan et al., 2024; Spiller et al., 2025; Theis et al., 2025; Wan et al., 2025)
High-protein diet (animal-based)	High animal protein, low fiber	↑ *Bacteroides*, *Clostridia*, *Fusobacterium*; ↓ butyrate producers	BCFAs (isobutyrate, isovalerate); ammonia; hydrogen sulfide; indoles; phenols; nitrosamines	Protein fermentation → toxic metabolites; epithelial stress	Impaired barrier function	Increased inflammation, colon damage	(Tomova et al., 2019; Pagliai et al., 2020; Duncan et al., 2021)
High-protein diet (plant-based)	Plant protein	↑ *Bifidobacterium*, *Lactobacillus*; ↓ pathogenic bacteria	Reduced toxic metabolites	Improved microbial balance	Protective effects	Reduced disease risk compared to animal protein	(Tomova et al., 2019)
High-fat diet	High fat intake	↑ *Firmicutes*/ *Bacteroidetes* ratio; ↑ *Bilophila wadsworthia*, *Alistipes*; ↓ SCFA producers	↑ Secondary bile acids (e.g., deoxycholic acid)	Bile acid-driven microbial selection; inflammation; insulin resistance	Barrier dysfunction, metabolic disruption	NAFLD, insulin resistance, systemic inflammation	(Guzior and Quinn, 2021; Du et al., 2025; Mamun et al., 2025; Tyagi and Kumar, 2025)

EVOO, as the MD’s cornerstone component, modulates microbial populations while serving as a substrate for the synthesis of cardioprotective and anti-inflammatory molecules. Olive-derived phenolics (hydroxytyrosol, tyrosol) and monounsaturated fats stimulate beneficial microbial populations (**Table 3**), reduce the *Firmicutes*/*Bacteroidetes* ratio, and help fortify barrier and immune functions (Garrido-Romero et al., 2025). Plant-enriched “Green-MD” variants and probiotic supplementation further optimize SCFA production, microbial diversity, and metabolic health, particularly benefiting populations at elevated risk for obesity, diabetes, and cancer (Perrone and D'Angelo, 2025).

### Vegetarian and vegan diets: plant-powered microbiomes and health

Vegetarian and vegan diets, by excluding meat (and, for vegans, all animal-derived foods), offer a bounty of plant fibers, complex carbohydrates, and polyphenols, but typically run low in fat and protein (Tomova et al., 2019; Losno et al., 2021; Soldan et al., 2024). These diets favor the proliferation of certain enterotypes that facilitate the fermentation of complex carbohydrates and correlate with high-fiber, plant-rich consumption (**Table 3**) (David et al., 2014; Verhoog et al., 2019; Losno et al., 2021; Soldan et al., 2024; Lotankar et al., 2025). Notably, SCFA benefits plateau in some Western populations due to adaptation effects, where excessive fiber intake may not linearly increase SCFA output (Losno et al., 2021; Trefflich et al., 2021; Wu et al., 2025). Additionally, the reported decrease in branched-chain fatty acid and ammonia production reflects lower protein fermentation and reduced generation of potentially toxic metabolites, such as p-cresol or indoxyl sulfate, on VD (Croci et al., 2021; Trefflich et al., 2021). Microbes convert dietary polyphenols into bioactive metabolites that stimulate the growth of beneficial taxa while suppressing pathobionts (David et al., 2014; Tomova et al., 2019; Rodriguez-Daza et al., 2021; Cano et al., 2024). These dietary patterns reduce BMI, cardiometabolic disease risk, and serum inflammatory markers (CRP, IL-6). They may lower colon cancer risk, though vitamin B12, vitamin D, and calcium supplementation remain essential to prevent deficiency-induced alterations in the microbiota (Croci et al., 2021; Losno et al., 2021).

### Dysbiosis and the perils of westernized nutrition

The Western diet epitomizes nutritional dysregulation through excessive saturated and trans fats, processed and red meats, refined grains, sugars, salt, and ultra-processed foods (UPFs), while depleting fiber, polyphenols, and whole foods like unprocessed fruits and vegetables, nuts, legumes, and whole grains, combined with food additives and low micronutrient density (Spiller et al., 2025; Theis et al., 2025). This dietary pattern results in profound loss of microbial diversity (**Table 3**) (Jangid et al., 2022; Spiller et al., 2025). Diminished SCFA production compromises epithelial integrity, increases intestinal permeability, and elevates circulating endotoxins (Dmytriv et al., 2024; Spiller et al., 2025; Theis et al., 2025) while amplifying TMAO, secondary bile acids, and pro-inflammatory metabolites linked to obesity, cardiovascular disease, IBD, and cancer (Simo and Garcia-Canas, 2020; Croci et al., 2021; Gill et al., 2022; Li J. et al., 2022; Soldan et al., 2024; Spiller et al., 2025; Wan et al., 2025). UPF additives further degrade *Bifidobacteria* and promote bacterial translocation, exacerbating metabolic endotoxemia (Dmytriv et al., 2024; Spiller et al., 2025).

### High-protein diets: microbial responses to amino acid abundance

Animal-derived high-protein diets direct an increase in proteolytic bacteria (e.g., *Bacteroides*, *Clostridia*, and *Fusobacterium*) while depleting butyrate-producers due to loss of fiber-fermenters and substrate limitation, generating branched-chain fatty acids (BCFAs) and toxic metabolites, including ammonia and hydrogen sulfide, indoles, phenols, and nitrosamines, that may compromise colonocyte integrity (Tomova et al., 2019; Pagliai et al., 2020; Duncan et al., 2021). An increase in BCFAs (e.g., isobutyrate, isovalerate) is generally considered less beneficial compared to SCFAs, and high concentrations may induce cellular stress and apoptosis in colonocytes, potentially impairing barrier function. However, plant-based protein tends to increase *Bifidobacterium* and *Lactobacillus* and reduce pathogenic bacteria, partly offsetting the negative impacts of animal protein fermentation (Tomova et al., 2019).

### High-fat diets and microbial dysbiosis

A high-fat diet shifts the *Firmicutes/Bacteroidetes* ratio, expanding pathobiont genera and reducing SCFA producers despite limited substrate availability. It leads to a decline in mucus-degrading, barrier-supporting bacteria, worsening gut permeability (Du et al., 2025; Mamun et al., 2025; Tyagi and Kumar, 2025). High-fat intake enhances bile acid synthesis and favors genotoxic secondary bile acids (e.g., deoxycholic acid), selecting for bile salt-resistant, pro-inflammatory taxa (e.g., *Bilophila wadsworthia*, *Alistipes*) and promoting systemic inflammation, insulin resistance, and NAFLD development (Guzior and Quinn, 2021; Du et al., 2025; Mamun et al., 2025; Tyagi and Kumar, 2025).

## Host gene-diet interactions: the molecular symphony

The symphony of metabolism unfolds through a molecular choreography between our genetic blueprint and dietary choices. Host gene-diet interactions represent the dynamic dialogue and bidirectional relationship in which an individual’s genetic architecture influences their physiological and metabolic responses to specific dietary components, ultimately shaping their microbiota, health, and aging trajectories. While the literature is replete with examples of host gene-diet interactions in disease, the role of microbial alterations and their feedback regulation of these interactions has not been systematically studied. The microbiota often shapes the host gene-diet interaction, and the profound impact of understanding these interactions lies in their immense therapeutic potential to revolutionize personalized medication, care, and health (Wu Q. et al., 2022; Virolainen et al., 2023; Farris et al., 2024). The host gene-diet interactions discussed in this section (**Table 4**) were selected on the basis of three criteria: (i) each polymorphism ranks among the most robustly replicated in genome-wide association or candidate gene studies for its corresponding disease phenotype; (ii) a well-documented interaction with a specific macronutrient, or dietary pattern, or nutrient status already exists in human cohorts or controlled intervention trials; and (iii) the interactions collectively span the disease categories most relevant to aging and the diet-microbiota-host gene triad, including metabolic, cardiovascular, neurological, and inflammatory pathways. This selection is intended necessarily as an illustrative rather than exhaustive one, and many additional loci undoubtedly participate in diet-responsive, microbiota-influenced phenotypes and await systemic characterization. Where evidence for microbiota involvement exists, whether direct or inferential, is noted explicitly.

**Table 4:** Gene–diet interactions in disease and metabolism.

Gene	Polymorphis m / Variant	Diet / Nutrient	Microbiota involvement	Key metabolites	Mechanism / pathway	Metabolic effects	Health outcomes	Disease association	References
TCF7L2	rs7903146 (risk allele T)	High-fiber diet	Fiber-fermenting microbiota (SCFA producers)	SCFAs	Impaired incretin signaling despite SCFA production	↑ insulin, proinsulin, C-peptide; impaired glucose regulation	Reduced protective effect of fiber	Type 2 diabetes, impaired glucose tolerance	(Grant et al., 2006; Hindy et al., 2012; Hindy et al., 2016; Ortega et al., 2017; Dietrich et al., 2019; Ferreira et al., 2019; Aboelkhair et al., 2021; Del Bosque-Plata et al., 2021; Fry et al., 2022; Duarte et al., 2025)
IRS1	rs2943641 (CC genotype)	High-carbohydrate, low-fat diet	diet-driven metabolic modulation	Glucose-derived metabolites	Enhanced IRS1–PI3K signaling under low-fat conditions	↑ insulin sensitivity, ↑ weight loss	Reversal of genetic insulin resistance	Obesity, insulin resistance	(Hoehn et al., 2008; Langlais et al., 2011; Qi et al., 2011; Qi et al., 2013; Brown and Walker, 2016; Bray et al., 2019; Li M. et al., 2022; Duarte et al., 2025; Toyoshima et al., 2025)
APOA5	rs662799, rs2266788	Dietary fat composition, carbohydrate intake, calcium intake	lipid metabolism–microbiota axis	Triglycerides (TG)	Altered lipid metabolism and TG regulation	↑ TG under low-fat diet; ↓ TG under high-carb diet; calcium-dependent modulation	Variable lipid response	Cardiovascular disease, metabolic syndrome	(Jiang et al., 2010; Ken-Dror et al., 2010; Hubacek, 2016; Lin et al., 2016; Kim et al., 2024)
ZPR-1	rs964184 C>G	Mediterranean vs low-fat diet	diet–lipid metabolism axis	Triglycerides	Differential postprandial lipid metabolism	Improved TG clearance under low-fat diet (G allele)	Optimized lipid profile in carriers	Cardiovascular disease, hyper-cholesterolemia	(Wojczynski et al., 2015; Delgado-Lista et al., 2016; Paquette et al., 2020; Alcala-Diaz et al., 2022)
MTHFR	C677T (TT, CT genotypes)	Folate intake; Mediterranean diet	Microbiota contributes to folate metabolism	Homocysteine	One-carbon metabolism regulation	↓ homocysteine (MD); ↑ risk with excess folate	Context-dependent benefit/risk	Stroke, cardiovascular disease	(Fowkes et al., 2000; Ashfield-Watt et al., 2002; Wernimont et al., 2012; Xuan et al., 2014; Liew and Gupta, 2015; Bennett et al., 2023; Zhang J. et al., 2025)
ABCA1	rs1883025 (T allele)	High-protein diet	Amino acid metabolic signaling pathways	HDL-related lipids	mTOR and AMPK modulation	Improved lipid transport	↓ ischemic stroke risk	Cardiovascular disease, AMD	(Li et al., 2014; Rajendran et al., 2018; Mockute et al., 2021; Peters et al., 2022; Lee JH. et al., 2025)
APOE	ϵ4 allele	High-fat diet, ketogenic diet, Mediterranean diet	Gut–brain axis (microbiota-derived metabolites)	Lipids, ketone bodies	Altered lipid transport, glucose hypometabolism	Improved cognition with acute fat intake; resistance to KD	Context-dependent cognitive effects	Alzheimer’s disease	(Petot et al., 2003; Barberger-Gateau et al., 2011; Hanson et al., 2013; Montagne et al., 2020; Yassine and Finch, 2020; Angelopoulou et al., 2021; Husain et al., 2021; Barisano et al., 2022; Hersant and Grossberg, 2022; Troutwine et al., 2022; Liu et al., 2024; Ivanich et al., 2025; Liu et al., 2025b; Pawlowska et al., 2025; Urich et al., 2025)
ATP7B	K832R variant	Copper intake	metal–microbiota interactions	Copper	Copper transport dysregulation	↑ free copper, oxidative stress	Improved with copper restriction	Alzheimer’s disease	(Hsu et al., 2018; McCann et al., 2019; Agarwal et al., 2022; Hureau, 2023; Sabalic et al., 2024)
TNFA	rs1800629 (-308G→A)	Mediterranean diet	Anti-inflammatory microbiota /metabolites (polyphenols, SCFAs)	Cytokines, triglycerides	Modulation of inflammatory signaling pathways	↓ inflammation, ↓ TG levels	Improved inflammato ry profile	Obesity, CVD, multiple sclerosis	(Dalziel et al., 2002; Joffe et al., 2010; Gomez-Delgado et al., 2014; Sureda et al., 2018; Leonska-Duniec et al., 2019; Marginean et al., 2019; Frye et al., 2024)
FTO	rs9939609, rs1421085	High-fat, high-sugar diets; Mediterranean diet	Gut–brain–microbiota axis (behavioral + metabolic)	Energy metabolites	Neural regulation of appetite and reward	↑ preference for calorie-dense foods	Obesity risk modulated by MD	Obesity	(Seral-Cortes et al., 2022; Poosri et al., 2024)
GSTM1	Gene deletion (null)	Phytochemical -rich diet (vegetables)	Microbial metabolism of phytochemicals	Antioxidant metabolites	Detoxification pathways	Enhanced benefit from phytochemicals	Reduced carcinogen ic risk	Cancer, vascular disease	(Lafuente et al., 1995; Lizard-Nacol et al., 1999; Piao et al., 2009; Saitou et al., 2018; DeBenedictis et al., 2024)
HMGCR	rs17238540 (T>G)	Fiber vs saturated fat intake	Fiber–microbiota–lipid axis	Lipids (TG)	Cholesterol synthesis regulation	Greater TG reduction with fiber; sensitivity to saturated fat	Improved lipid control (G allele)	Cardiovascular disease	(Polisecki et al., 2008; Freitas et al., 2010; Xu et al., 2015)
LDLR	p.Glu179Met and other variants	Dietary cholesterol	cholesterol metabolism axis	LDL cholesterol	Lipoprotein clearance	Personalized cholesterol response	Improved manageme nt with tailored diet	Familial hyper-cholesterolemia	(Pussadhamma et al., 2024)
PCSK9	Functional variants	High-fat diet	Lipid metabolism–microbiota axis	Lipids	Regulation of LDL receptor degradation	Altered lipid metabolism under high-fat intake	Impacts therapy response	Cardiovascular disease	(Nemeth et al., 2023; Bao et al., 2024)
CETP	rs3764261	Mediterranean diet	Polyphenol– microbiota metabolism	HDL cholesterol, triglycerides	Lipid transfer modulation	↑ HDL, ↓ TG (A allele carriers)	Improved lipid profile	Cardiovascular disease	(Qi et al., 2015; Wuni et al., 2022)

### Gene-diet interactions that influence metabolism

#### TCF7L2 and dietary fiber: the paradox of genetic susceptibility

The transcription factor 7-like 2 (TCF7L2) gene occupies a central position in Type 2 diabetes mellitus pathogenesis, representing the most robust genetic risk factor identified through GWAS studies. TCF7L2 functions as a central transcriptional regulator of the adipocyte metabolic program by directly regulating genes involved in glucose and lipid metabolism (Grant et al., 2006; Ortega et al., 2017). Recent investigations have unveiled a remarkable paradox: while dietary fiber is traditionally considered protective against diabetes pathophysiology, carriers of the TCF7L2 risk alleles demonstrate increased susceptibility when consuming high-fiber diets, with increased insulin, proinsulin, and C-peptide responses, although the exact mechanism remains unknown. The rs7903146 polymorphism, with a counterintuitive relationship, appears to stem from impaired incretin signaling in T-allele carriers, in whom SCFAs produced through microbial fermentation of fiber fail to provide their typical protective effects on glucose homeostasis (Hindy et al., 2012). It also has a profound impact on non-diabetic older adults with the risk allele, who show an increased risk for glucose intolerance (Hindy et al., 2012; Hindy et al., 2016; Dietrich et al., 2019; Ferreira et al., 2019; Aboelkhair et al., 2021; Del Bosque-Plata et al., 2021; Fry et al., 2022; Duarte et al., 2025).

#### IRS1 and macronutrient composition: personalized insulin sensitivity

The insulin receptor substrate 1 (IRS1) gene is a fascinating example of how genetic variation can fundamentally alter the outcomes of dietary interventions. The rs2943641 polymorphism demonstrates profound interactions with macronutrient composition, particularly the carbohydrate-to-fat ratio (Brown and Walker, 2016; Duarte et al., 2025). Recent research also reveals that deletion of IRS-1 leads to growth failure and insulin resistance in animal models, highlighting its critical role in metabolic regulation (Toyoshima et al., 2025). Individuals carrying the CC genotype, traditionally associated with increased resistance, paradoxically demonstrated superior improvements in insulin sensitivity and weight loss when consuming high-carbohydrate, low-fat diets compared to non-carriers (Qi et al., 2011; Qi et al., 2013; Bray et al., 2019). This landmark finding from the POUNDS Lost Trial suggests that CC carriers may experience enhanced IRS-1-associated PI3K activity under low-fat conditions, effectively reversing their genetic predisposition to insulin resistance (Hoehn et al., 2008; Langlais et al., 2011; Qi et al., 2011; Bray et al., 2019; Li M. et al., 2022). A direct link to altered microbiota in this interaction has not been elucidated.

### Cardiovascular health: the lipid metabolism network

#### APOA5 and dietary fat composition for triglyceride management

Apolipoprotein A-V (APOA5) is among the most potent genetic determinants of plasma triglyceride levels, with variants demonstrating significant interactions with dietary fat composition. The rs662799 and rs2266788 polymorphisms create differential responses to macronutrient intake, fundamentally altering cardiovascular risk profiles. Recent research has demonstrated that different APOA5 SNPs and haplotypes exhibit distinct effects on triglyceride levels and cardiovascular disease risk, with carriers of APOA5 minor alleles showing paradoxical responses to dietary fat modification. While low-fat intake typically reduces triglyceride levels, minor allele carriers demonstrate increased plasma triglycerides under these conditions. Several SNPs in the APOA5, BUD13, CETP, and LIPA genes independently affect the prevalence of metabolic syndrome and do so through complex gene-diet interactions (Ken-Dror et al., 2010; Hubacek, 2016; Lin et al., 2016; Kim et al., 2024). APOA5 is predominantly expressed in the liver and is an important determinant of plasma TG levels. Interestingly, low-fat intake increases plasma TG levels, whereas high-carbohydrate intake lowers them in individuals carrying the minor alleles. Individuals who are carriers of rs662799 and have low calcium intake display higher plasma TG concentrations than those with moderate calcium intake (Jiang et al., 2010). How this gene-diet pair interacts with the microbiota to shape the unique phenotypic outcome is an open question.

##### ZPR-1 and Mediterranean diet for postprandial metabolism

The zinc finger protein ZPR-1 gene rs964184 C>G polymorphism explored in the CORDIOPREV study has demonstrated that the rs964184 polymorphism creates differential responses to Mediterranean versus low-fat dietary patterns (Delgado-Lista et al., 2016; Alcala-Diaz et al., 2022). While Mediterranean diets are traditionally considered optimal for cardiovascular health, carriers of the G allele demonstrate superior postprandial triglyceride responses to low-fat diets when compared to Mediterranean dietary patterns (Wojczynski et al., 2015). G-allele carriers exhibit enhanced triglyceride clearance mechanisms under low-fat conditions, effectively normalizing their lipid profiles to match those of non-carriers to mitigate cardiovascular disease risk and hypercholesterolemia (Paquette et al., 2020; Alcala-Diaz et al., 2022). No direct connection to microbiome alteration is proposed in this case.

### One-carbon metabolism and cardiovascular health

#### MTHFR and folate status: the double-edged sword

The methylenetetrahydrofolate reductase (MTHFR) gene plays a central role in one-carbon metabolism, and MTHFR polymorphisms that reduce enzyme efficiency are significantly associated with cardiovascular disease risk. The C677T polymorphism modulates the dynamic interactions between folate intake and cardiovascular health. Recent research has shown that Mediterranean diet intake reduces homocysteine concentrations specifically in TT and CT genotype carriers but not in CC individuals. The TT genotype significantly elevates cerebrovascular stroke risk in populations with suboptimal folate status (Ashfield-Watt et al., 2002; Bennett et al., 2023). However, contemporary research has unveiled a concerning paradox: excessive folate supplementation in individuals with existing cardiovascular disease may increase mortality risk, particularly in MTHFR variant carriers. This finding suggests that the protective effects of folate depend heavily on baseline health status and genetic background, necessitating personalized folate recommendations (Fowkes et al., 2000; Ashfield-Watt et al., 2002; Dedoussis et al., 2004; Wernimont et al., 2012; Xuan et al., 2014; Liew and Gupta, 2015; Bennett et al., 2023; Zhang J. et al., 2025). It is possible that the effect of the Mediterranean diet on the folate-producing *Bifidobacterium* and *Lactobacillus* may explain the effect, although the direct mechanism has not been elucidated.

#### ABCA1 and dietary protein for cholesterol management

The ATP-binding cassette transporter A1 (ABCA1) gene rs1883025 polymorphism, traditionally associated with reduced HDL cholesterol levels and age-related macular degeneration (AMD), shows a remarkable protective effect when combined with a high-protein dietary pattern. Carriers of the T allele exhibit significantly reduced ischemic stroke risk when consuming protein-rich diets, suggesting that dietary protein may enhance ABCA1 activity through modulation of mTOR and AMPK signaling pathways. The rs1883025 polymorphism is a meaningful genetic marker, but its clinical significance is nuanced (Li et al., 2014; Rajendran et al., 2018; Mockute et al., 2021; Peters et al., 2022; Lee JH. et al., 2025). Additionally, contributions from the microbiota have not been studied.

### Neurological health: the brain-diet-gene axis and neurodegenerative disease

#### APOE and dietary fat: cognitive fuel preferences

The apolipoprotein E (APOE) gene remains the most significant genetic determinant of Alzheimer’s disease risk, with recent research demonstrating that the ϵ4 allele confers up to a 10-fold increased susceptibility in homozygous carriers (Montagne et al., 2020; Barisano et al., 2022; Troutwine et al., 2022). These carriers also exhibit a unique dietary fat preference, which creates fundamental alterations in brain metabolism. The APOE ϵ4 allele is associated with weakening neuronal repair, overwhelming amyloid plaque formation, and heightened oxidative stress. Older APOE ϵ4 carriers with cognitive impairment exhibit improved cognitive function and normalized plasma biomarkers following acute high-fat meal consumption, while non-carriers show cognitive deterioration under identical conditions (Petot et al., 2003). This differential response suggests that APOE4 carriers may require alternative brain fuel sources, possibly due to glucose hypometabolism characteristic of the variant. However, the relationship is complex, as APOE4 carriers also demonstrate resistance to ketogenic diet interventions, highlighting the intricate nature of host gene-diet interactions in neurological health (Hersant and Grossberg, 2022; Pawlowska et al., 2025; Urich et al., 2025). This resistance may reflect the altered lipid transport and clearance functions associated with the ϵ4 variant, where conventional ketogenic approaches fail to provide optimal brain fuel delivery (Barberger-Gateau et al., 2011; Hanson et al., 2013; Yassine and Finch, 2020; Angelopoulou et al., 2021; Husain et al., 2021; Liu et al., 2024; Ivanich et al., 2025; Pawlowska et al., 2025; Urich et al., 2025). The Mediterranean diet has also been found to be more effective in modulating dementia-related metabolites in APOE4 homozygotes (Liu et al., 2025b). A significant microbiota interaction may be envisioned as *APOE* genotype status shapes the baseline gut microbiome (Tran et al., 2019; Parikh et al., 2020). Carriers of the ϵ4 allele naturally exhibit a lower abundance of SCFA-producing taxa and an increased prevalence of pro-inflammatory, lipopolysaccharide (LPS)-producing Gram-negative bacteria (Tran et al., 2019). However, the exact molecular interaction has not been worked out.

#### ATP7B and copper homeostasis: metal-gene interactions in neurodegeneration

The copper-transporting P-type ATPase ATP7B gene has emerged as a critical modulator of Alzheimer’s disease risk by influencing copper homeostasis. Recent investigations have revealed that polymorphisms in ATP7B, particularly the K832R variant, create profound interactions with dietary copper intake. Carriers of loss-of-function ATP7B variants demonstrate elevated serum free copper levels, which correlate with increased amyloid-β deposition and enhanced oxidative stress in brain tissue. Remarkably, dietary copper restriction can significantly ameliorate these pathological changes, effectively normalizing copper homeostasis in genetically susceptible individuals (Hsu et al., 2018; McCann et al., 2019; Agarwal et al., 2022; Hureau, 2023; Sabalic et al., 2024). Specific microbiota interaction with this diet-host gene pair has not been elucidated.

### Inflammatory pathways and diet

#### TNFA and Mediterranean diet: anti-inflammatory genetic modulation

The tumor necrosis factor-alpha (TNFA) gene is a critical node in the regulation of the inflammatory pathway. Individuals with the -308G→A promoter polymorphism (rs1800629) exhibit higher BMI, higher leptin levels, and disproportionately higher levels of soluble TNFR2, making them genetically predisposed to increased risk of obesity, CVD, and multiple sclerosis (Dalziel et al., 2002; Joffe et al., 2010; Sureda et al., 2018; Leonska-Duniec et al., 2019; Marginean et al., 2019). Recent clinical trials have demonstrated that Mediterranean dietary patterns, through their abundant anti-inflammatory compounds, including polyphenols and omega-3 fatty acids, can effectively modulate the inflammatory consequences of TNFA genetic variation, by significantly reducing the triglyceride levels and inflammatory markers (Gomez-Delgado et al., 2014). Primate model-based research has elucidated that Mediterranean diet consumption significantly downregulates pro-inflammatory genes, including cyclin-dependent kinase 14 (CDK14), while upregulating anti-inflammatory pathways (Gomez-Delgado et al., 2014; Sureda et al., 2018; Frye et al., 2024). However, the direct involvement of microbiota interaction has not been addressed in these studies.

### Obesity and energy homeostasis

#### FTO and dietary composition: the brain-gut-gene axis

The fat mass and obesity-associated (FTO) gene represents a major genetic determinant of obesity risk, with sophisticated interactions between FTO variants and dietary macronutrient preferences that influence both metabolic outcomes and neurological function (Poosri et al., 2024). The rs9939609 and rs1421085 polymorphisms significantly influence dietary behavior, with risk-allele carriers showing an elevated preference for high-sugar and high-fat foods. These variants affect the medial prefrontal cortex, the brain region controlling appetite regulation, creating sustained alterations in food preference patterns through modified neural reward pathways. Intriguingly, the FTO genotype may also act upstream of the gut-brain axis, with risk alleles influencing gut microbial ecology under specific dietary conditions (Sun et al., 2019; Zheng et al., 2022). Under high-fat feeding, the associated dysbiotic microbiota may generate altered neuroactive and metabolic signals that feedback through vagal and humoral pathways, disrupting central satiety pathways, and potentially reinforcing central hyperphagia and weight gain, though the mechanistic chain from genotype to microbiota to neural reward circuitry remains to be directly demonstrated (Karra et al., 2013; Torres-Fuentes et al., 2017).

Notably, Mediterranean dietary patterns can effectively counteract the obesity-promoting effects of FTO variants, suggesting enhanced protective benefits in genetically susceptible individuals (Seral-Cortes et al., 2022; Poosri et al., 2024).

### Detoxification and cancer prevention

#### GSTM1 and phytochemical protection for cancer prevention

The glutathione S-transferase mu 1 (GSTM1) gene deletion polymorphism creates significant interactions with dietary phytochemical intake that influence cancer risk, including melanoma, lung, and colon cancer, as well as other conditions like vascular remodeling and atherosclerosis. Recent research has shown that the protective effects of vegetable consumption are particularly pronounced in individuals with the GSTM1-null genotype. This differential effect suggests that heat-labile phytochemicals may be particularly important for individuals with compromised detoxification capacity (Lafuente et al., 1995; Lizard-Nacol et al., 1999; Piao et al., 2009; Saitou et al., 2018; DeBenedictis et al., 2024).

#### HMGCR and lipid-lowering interventions

The 3-hydroxy-3-methylglutaryl-coenzyme A reductase (HMGCR) gene demonstrates significant interactions with both dietary and pharmacological interventions (Polisecki et al., 2008). The T>G polymorphism (rs17238540) creates differential responses to dietary fiber and saturated fat intake that influence cardiovascular disease risk. Carriers of the G allele demonstrate enhanced benefits from dietary fiber consumption, exhibiting greater reductions in serum triglycerides compared to TT individuals, while showing increased sensitivity to saturated fat intake (Freitas et al., 2010; Xu et al., 2015).

Although possible, the interactions and roles of microbiota in cancer predisposition have not been investigated in the above studies.

### Emerging gene-diet interactions in cardiovascular health

#### LDLR and dietary cholesterol: familial hypercholesterolemia management

Recent research on familial hypercholesterolemia reveals complex interactions between LDL receptor (LDLR) variants and dietary interventions. Individuals with specific LDLR mutations, including the novel p.Glu179Met variant identified in Thai populations, benefit from tailored dietary approaches that consider genetic effects and therapeutic interventions. These discoveries demonstrate that genetic variation in cholesterol metabolism creates personalized dietary requirements (Pussadhamma et al., 2024).

#### PCSK9 and dietary fat: metabolic regulation and therapeutic implications

Proprotein convertase subtilisin/kexin type 9 (PCSK9) functions as a critical cholesterol homeostasis regulator with significant dietary fat interactions. High-fat diets downregulate hepatic PCSK9 expression, triggering compensatory mechanisms that affect metabolic outcomes and therapeutic responses. PCSK9 knockout mice exhibit altered hepatic lipid profiles under high-fat feeding, indicating that dietary composition significantly influences the consequences of genetic variation in cholesterol-regulating pathways, with implications for PCSK9 inhibitor therapies (Bao et al., 2024).

#### CETP and Mediterranean diet: HDL optimization strategies

Cholesteryl ester transfer protein (CETP) variants interact significantly with dietary patterns, particularly Mediterranean diets. The rs3764261 polymorphism creates differential responses to Mediterranean versus low-fat diets. Minor A-allele carriers demonstrate superior HDL cholesterol responses and lower triglycerides after 12 months of Mediterranean diet adherence compared to conventional low-fat patterns. This interaction is mediated by Mediterranean diet polyphenols and monounsaturated fats, which preferentially benefit specific CETP genetic variants (Qi et al., 2015; Wuni et al., 2022).

Across the gene-diet interactions reviewed above, microbiota emerges as a plausible, and in some cases partially documented mediator; yet its precise contribution remains largely unexplored. Many of these interactions involve dietary substrates that are processed by gut microbial communities before reaching host metabolic targets, suggesting that the microbiota may modulate the phenotypic consequences of genetic variation in ways that current dyadic study designs cannot fully capture. Future investigations that specifically incorporate microbiome profiling and manipulation alongside genetic and dietary variables will be essential for advancing from pairwise associations to a fully integrated understanding of the diet-microbiota-host gene triad in health, disease, and aging.

## Sex differences in the diet-microbiota-host gene triad

The gut microbiome composition, immune-microbiome crosstalk, and dietary metabolism all exhibit significant sex-specific patterns that modulate the diet-microbiota-host gene triad (Hernandez-Acosta et al., 2025). Sex hormones, such as estrogens and androgens, directly shape microbial community structure throughout the lifespan (Shin et al., 2019; d'Afflitto et al., 2022). Estrogen is associated with greater relative *Lactobacillus* abundance and higher overall microbial diversity in premenopausal women than in men of similar age (Ravel et al., 2011; Santos-Marcos et al., 2018). The decline in circulating estrogens following menopause correlates with a shift towards more pro-inflammatory microbiome composition, a change that may contribute to the accelerated bone loss and increased cardiovascular risk characteristics of the postmenopausal period (Lee K. et al., 2025; Wang et al., 2025). In males, higher circulating testosterone is associated with a reduced relative abundance of the *Bacteroidetes* taxa and altered SCFA profiles, suggesting androgen-driven microbial configurations create distinct metabolic landscapes in men and women (Santos-Marcos et al., 2018; Matsushita et al., 2022). Sex-specific immune responses to microbial metabolites are also well documented, in which women generally mount stronger innate and adaptive immune responses that interact with microbiome-derived signals to generate differential susceptibility to autoimmune and inflammatory diseases (Klein and Flanagan, 2016; Ortona et al., 2019). Gene-diet interactions are equally subject to sex-specific modulation; for example, the effect size of the FTO *rs9939609* obesity risk allele differs between men and women, and the cognitive response to dietary fat in APOE4 carriers shows sex-dependent outcomes (Ben Halima et al., 2018; Park and Choi, 2023). Collectively, these observations underscore the importance of treating sex as a fundamental biological variable in microbiome and nutritional genomics research. Many of the host gene-diet examples discussed in this review remain to be validated in appropriately sex-stratified cohorts, and future triad studies should be designed with sufficient power to detect sex-by-diet-by-microbiota interactions.

## The non-bacterial microbiome: mycobiome, virome, and archaeome

The microbial ecosystem of the human gut extends well beyond bacteria. The gastrointestinal tract harbors distinct communities of fungi, viruses, and archaea, collectively forming a multi-kingdom ecosystem whose contributions to health, disease, and aging remain considerably less well characterized than those of the bacterial microbiome (Barrera-Vazquez and Gomez-Verjan, 2020). These non-bacterial kingdoms are, nonetheless, dynamic participants in the diet-microbiota-host gene triad: their community structures are sensitive to dietary inputs, shaped by host genetic and immune programs, and subject to significant remodeling across the lifespan (Barrera-Vazquez and Gomez-Verjan, 2020; Chin et al., 2020; Zhang et al., 2022; Chue et al., 2026). The sections below briefly outline the mycobiome, virome, and archaeome in the context of the triad.

### The mycobiome

The human gut mycobiome comprises a low-abundance but functionally important eukaryotic community, with genera such as *Saccharomyces*, *Candida*, *Malassezia*, and *Cladosporium* most frequently reported in adult cohorts (Barrera-Vazquez and Gomez-Verjan, 2020; Chin et al., 2020; Zhang et al., 2022; Chue et al., 2026). Fungal populations are highly diet-responsive. Carbohydrate-rich patterns are associated with an increased relative abundance of *Candida albicans*, whereas higher protein and fat intake tends to deplete *Candida* and favor enrichment of *Saccharomyces* species (Hoffmann et al., 2013). Fungi also engage in extensive cross−talk with the bacterial microbiota through mixed−species biofilms and metabolic exchange. Under conditions of antibiotic−induced bacterial depletion, opportunists such as *C. albicans* can expand, while beneficial yeasts such as *Saccharomyces boulardii* secrete proteases that degrade bacterial toxins and help attenuate mucosal inflammation (Ji et al., 1999; Erb Downward et al., 2013). Aging is associated with reduced fungal diversity and a relative expansion of pathobiontic Candida species, which engage C−type lectin receptors such as Dectin−1 and can contribute to barrier disruption and systemic inflammaging (Jayaraman et al., 2026).

### The virome

The gut virome is dominated numerically by bacteriophages (the phageome), which are among the most abundant biological entities in the human body (Barrera-Vazquez and Gomez-Verjan, 2020; Howard et al., 2024; Chue et al., 2026). Phages act as ecological “rheostats” by selectively lysing bacterial hosts and, in the lysogenic state, transferring functional genes that remodel bacterial metabolic capacity and fitness (Lathakumari et al., 2025). Virome composition changes markedly across the lifespan: diversity is low at birth, increases during the first two years of life, and then gradually contracts as bacterial diversity expands, eventually stabilizing in adulthood. In older age, virome diversity tends to decline again, in parallel with waning barrier integrity, whereas centenarian cohorts exhibit a highly diverse phageome characterized by elevated lytic activity and enrichment of phages carrying auxiliary metabolic functions thought to support mucosal integrity and resistance to pathobionts (Gregory et al., 2020; Johansen et al., 2023; James et al., 2024). As with bacterial communities, the virome is highly sensitive to diet; Western, low-fiber, high−sugar patterns, gluten−free diets, and malnutrition all drive rapid and reproducible remodeling of the phage community structure (Howard et al., 2024; Lathakumari et al., 2025).

### The archaeome

The gut archaeome is dominated by methanogenic archaea, particularly *Methanobrevibacter smithii*, which occupies a distinct metabolic niche within the intestinal ecosystem (Samuel et al., 2007). Methanogens participate in syntrophic cross−kingdom networks with anaerobic bacteria by consuming molecular hydrogen and carbon dioxide produced during bacterial fermentation of complex carbohydrates and converting them into methane (Stams and Plugge, 2009; Hansen et al., 2011). This removal of hydrogen prevents accumulation of fermentation by−products and increases the efficiency of bacterial polysaccharide degradation and SCFA production (Hansen et al., 2011). Methane output, in turn, influences host physiology: higher intestinal methane levels are associated with slower transit and constipation−predominant bowel habits (Attaluri et al., 2010). Archaeal abundance is strongly shaped by diet; patterns rich in complex carbohydrates and fermentable fiber promote methanogen expansion by sustaining high bacterial hydrogen flux, whereas high−protein diets are generally associated with lower *M. smithii* prevalence (Hoffmann et al., 2013). Metagenomic analyses of centenarian microbiomes indicate that healthy longevity is associated with the retention of archaeal genes involved in coenzyme M and F420 biosynthesis, pathways that support metabolic homeostasis and protect against oxidative stress (Wu et al., 2019; Chen S. et al., 2024).

## Unresolved questions, technical hurdles, limitations, and future directions

Despite the breadth of literature summarized in this review, the diet-microbiota-host-gene triad remains an evolving framework with several important unanswered questions, methodological constraints, and translational gaps that should inform and guide future research.

### Inter-individual variability and population specificity

A central challenge in nutritional and microbiome research is marked by the heterogeneity in individual responses to dietary interventions. Outcomes are shaped by baseline microbiota composition, long-standing dietary habits, host genetic background, physical activity, age, sex, and ethnicity, among other factors (Gupta et al., 2017; Mills et al., 2019; Liang et al., 2026). Moreover, most gene-diet interaction studies to date have been conducted in cohorts of predominantly European ancestry, limiting the generalizability of their findings. Distinct, population-specific microbiome signatures, driven by geography, traditional dietary patterns, environmental exposures, and genetic ancestry, mean that dietary recommendations derived from one population cannot be assumed to apply uniformly to others (Kapellou et al., 2025). Future studies will need to deliberately recruit ethnically and geographically diverse cohorts and analyze diet-microbiota-gene interactions within these specific population contexts rather than extrapolating from a narrow reference group (Gupta et al., 2017; Mills et al., 2019; Liang et al., 2026).

### Causality and study design limitations

The majority of human evidence linking dysbiosis to aging and age-related diseases is observational and cross-sectional, which inherently limits causal inference. Such studies are often subject to substantial confounding from variables including polypharmacy (notably antibiotic and proton pump inhibitor use), stool form and transit time, and physical exercise, all of which can alter microbiome composition and contribute to poor reproducibility across independent cohorts (Falony et al., 2016; Zhernakova et al., 2016). In addition, widely used 16S rRNA gene surveys lack the taxonomic and functional resolution needed to distinguish species- or strain-level differences that may be critical for metabolite production and host interactions. Underscoring the need for broader adoption of deep shotgun metagenomics and metatranscriptomics in triad research (Knight et al., 2018).

### Translational bottlenecks from preclinical models

Rodent models remain indispensable for establishing mechanistic links between diet, microbiota, host pathways, and aging phenotypes. However, translating these findings to humans is extremely difficult, as mice and humans differ in key anatomical, immunological, and metabolic features (Beura et al., 2016). For example, ovariectomizedmouse models show increased gut β-glucuronidase activity, whereas postmenopausal women exhibit a depletion of this enzyme, illustrating that even carefully designed preclinical models can yield outcomes that diverge from clinical observations and contradict clinical realities (Cross et al., 2024; Walton and Abais-Battad, 2026). To bridge this gap, future research should emphasize longitudinal human cohort studies, Mendelian randomization approaches, and rigorously controlled dietary intervention trials with pre−specified microbiome and host endpoints.

### Precision nutrition and multi−omics integration

The emerging field of precision geronutrition, which integrates genomics, metagenomics, metabolomics, transcriptomics, and proteomics, offers a promising route toward truly individualized dietary strategies for optimizing healthspan (Park et al., 2026). A key objective is to modulate core aging pathways, including nutrient-sensing networks such as mTOR, AMPK, and sirtuins, and mitochondrial function, through targeted dietary and microbiome interventions. For instance, caloric restriction and targeted bioactive phytochemicals (including curcumin and epigallocatechin gallate) can attenuate chronically elevated mTORC1 activity and activate sirtuins, thereby promoting autophagy and delaying cellular senescence. Machine−learning approaches such as the *Manoka* tool integrate clinical, demographic, and multi−omics datasets to model complex bacterial co−abundance networks, improve the accuracy of biological age predictions, and support the design of personalized dietary recommendations for healthy longevity (Aldriwesh et al., 2026). To translate these concepts into practice, structured frameworks such as the PRIME roadmap have been proposed. PRIME comprises five phases: Profiling (using metagenomic and epigenetic aging clocks), Reviewing (integrating host genetic variants), Identifying (pinpointing targeted metabolic deficits), Mapping (designing culturally and contextually adapted precision nutrition plans), and Evaluating (longitudinal multi−omics monitoring for safety and efficacy). Implementing such frameworks will require systematic integration of host genetic information with microbiota functional profiling and metabolic readouts to generate clinically actionable precision nutrition recommendations. Likewise, the clinical deployment of microbiome−based therapies, including fecal microbiota transplantation (FMT), synbiotics, and postbiotics, should rest on robust evidence of causal efficacy from well−designed randomized controlled trials in aging−related conditions before routine adoption (Aldriwesh et al., 2026).

## Conclusion

**Figure 1:**
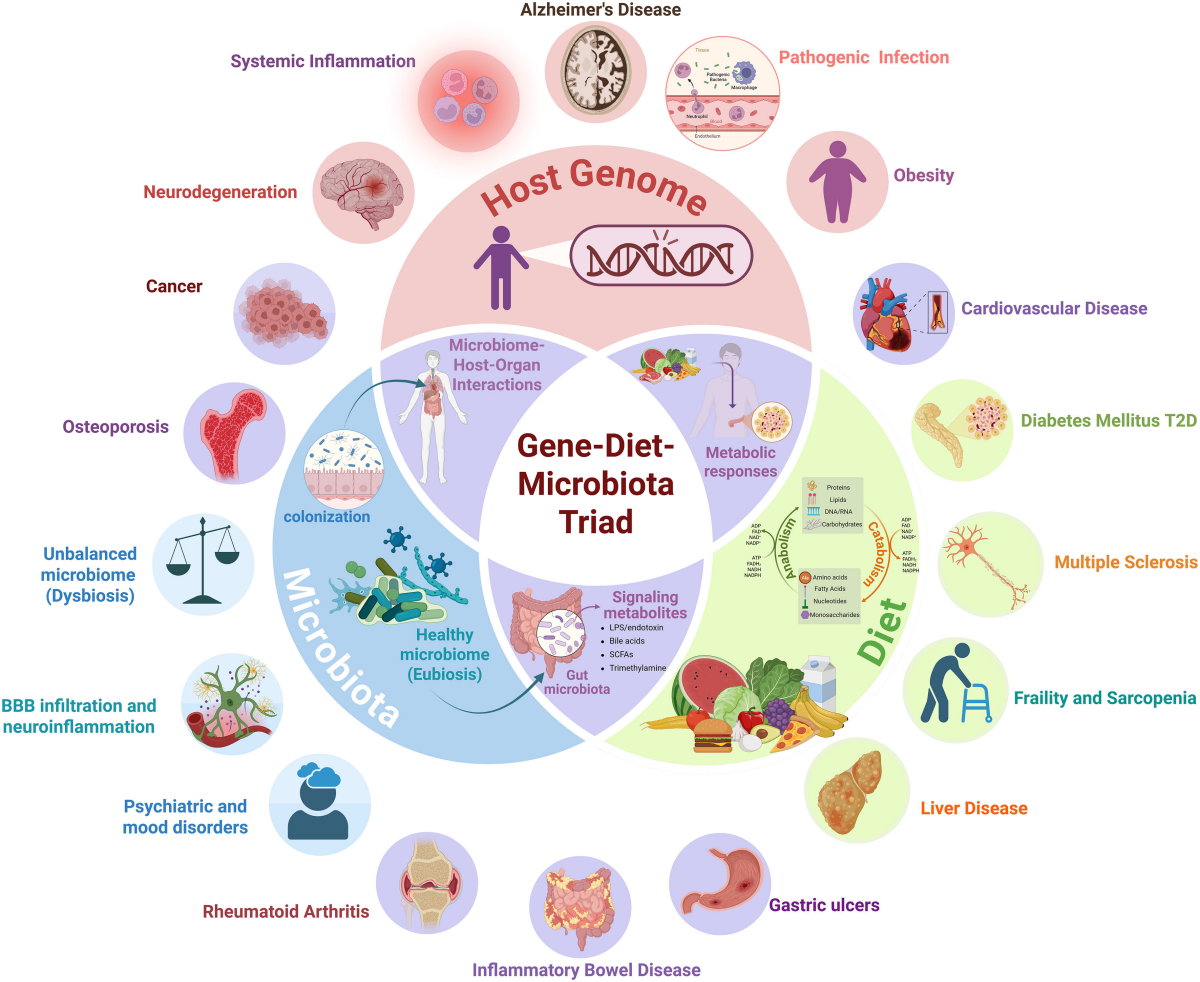
The Diet-Microbiota-Host Gene Triad in Human Physiology, Health and Disease. This schematic provides a high−level overview of the three main components of the triad: host genome, dietary patterns, and gut microbiota, and their bidirectional relationships. The central triangle emphasizes that genes, diet, and microbiota act together rather than independently, while the overlapping regions indicate, in simplified form, key interaction axes (for example, diet-microbiota metabolite production and microbiota-host organ communication). Peripheral labels group representative disease domains (metabolic, inflammatory, neurodegenerative, neoplastic, and age−related decline) that can arise when this triad is chronically perturbed. The figure is intended as a conceptual visual guide; detailed mechanistic links, specific metabolites, and points of age−related disruption are described in the main text rather than depicted exhaustively here.

The diet-microbiota-host gene triad is the pinnacle of a successful evolutionarily retained symbiotic partnership that benefits both the host and the microbiome. Health is not dictated by genes, diet, or microbiota in isolation, but by their continuous and dynamic interplay. This triad represents a co-regulated network that underlies metabolic homeostasis, immune balance, and organismal function across the lifespan. Any imbalance in either triadic counterpart has long-standing consequences for the host’s health (**Figure 1**). In this review, we have explored these interactions and their effects on physiology, aging, and disease. However, despite the breadth of our current knowledge, this framework remains incomplete.

The complexity of these ever-changing relationships and interactions, spanning spatial heterogeneity, temporal dynamics, environmental variability, and inter-individual differences across cultures, implies that many layers remain unresolved, including context-specific microbial functions, gene-environment interactions beyond diet, and causal mechanisms linking dysbiosis to disease.

Moving forward, research and clinical practice in aging and age-related diseases need to adopt this framework and consider the interplay among genetic variation, dietary patterns, and microbiome composition, and their interactions, as an integral stratification underlying health outcome. This has direct implications for medical research, especially in precision medicine, wherein therapeutic interventions, dietary guidelines, and public health policies can be tailored to preserve or restore the symbiotic balance. Ultimately, understanding and leveraging the diet-microbiota-host gene axis offers a path not only to treating diseases but also to maintaining resilience and building lifestyles that extend health span in an aging population worldwide.
